# Identification of key genes and pathways associated with resting mast cells in meningioma

**DOI:** 10.1186/s12885-021-08931-0

**Published:** 2021-11-12

**Authors:** Hui Xie, Ce Yuan, Xiao-hui Ding, Jin-jiang Li, Zhao-yang Li, Wei-cheng Lu

**Affiliations:** 1 Department of Histology and Embryology, College of Basic Medicine, Shenyang Medical College, Shenyang, Liaoning China; 2 Graduate Program in Bioinformatics and Computational Biology, University of Minnesota, Minneapolis, USA; 3 Department of Neurosurgery, General Hospital of Northern Theater Command, Shenyang, Liaoning China; 4 Department of Laboratory Animal Center, China Medical University, Shenyang, Liaoning China; 5 Department of Neurosurgery, First Affiliated Hospital of China Medical University, Shenyang, Liaoning China

**Keywords:** Meningioma, Resting mast cells, Differentially expressed genes, Pathways, miRNAs

## Abstract

**Background:**

To identify candidate key genes and pathways related to resting mast cells in meningioma and the underlying molecular mechanisms of meningioma.

**Methods:**

Gene expression profiles of the used microarray datasets were obtained from the Gene Expression Omnibus (GEO) database. GO and KEGG pathway enrichments of DEGs were analyzed using the ClusterProfiler package in R. The protein-protein interaction network (PPI), and TF-miRNA- mRNA co-expression networks were constructed. Further, the difference in immune infiltration was investigated using the CIBERSORT algorithm.

**Results:**

A total of 1499 DEGs were identified between tumor and normal controls. The analysis of the immune cell infiltration landscape showed that the probability of distribution of memory B cells, regulatory T cells (Tregs), and resting mast cells in tumor samples were significantly higher than those in the controls. Moreover, through WGCNA analysis, the module related to resting mast cells contained 158 DEGs, and KEGG pathway analysis revealed that the DEGs were dominant in the TNF signaling pathway, cytokine-cytokine receptor interaction, and IL-17 signaling pathway. Survival analysis of hub genes related to resting mast cells showed that the risk model was constructed based on 9 key genes. The TF-miRNA- mRNA co-regulation network, including MYC-miR-145-5p, TNFAIP3-miR-29c-3p, and TNFAIP3-hsa-miR-335-3p, were obtained. Further, 36 nodes and 197 interactions in the PPI network were identified.

**Conclusion:**

The results of this study revealed candidate key genes, miRNAs, and pathways related to resting mast cells involved in meningioma development, providing potential therapeutic targets for meningioma treatment.

**Supplementary Information:**

The online version contains supplementary material available at 10.1186/s12885-021-08931-0.

## Background

Meningioma, originating from meningothelial cells, is the most common primary brain tumor, accounting for 35–40% of central nervous system (CNS) tumors, with an incidence of approximately 2 in 100,000 adults [1, 2]. There is an about 3:1 female predominance of meningioma due to the role of estrogen [3, 4]. Meningioma is often associated with headaches, imbalances, impaired vision, and other neurological problems that can leave the patient very weak [5]. According to the WHO’s classification of nervous tissue tumors, meningiomas belong to the meningothelial-cell tumors of the meninges, and are classified into grades I, II and III. Treatment for meningioma encasing crucial neural and vascular structures, and for more aggressive histological types such as anaplastic (grade III), may be very challenging. Although most meningioma are classified as WHO grade I (85–95%), and considered as benign solid tumors, the tumors can recur even after complete resection, and the recurrence rate in these patients have been reported to be 7.5% or 9.3% in 10 and 20 years, respectively [6, 7]. It is therefore of great value to explore the molecular mechanism underlying the development of meningioma, as it will aid in disease treatment and patient prognosis improvement.

Generally, the development and growth of a tumor usually requires the appropriate microenvironment, as well as genetic/molecular changes in the tumor cell. An increasing number of evidence reports that the malignant phenotype of tumor is influenced by the tumor-related microenvironment [8–10]. The tumor-related microenvironment consists of tumor immune cell types, density, and localization, and accumulating evidence has shown correlations between immune cell infiltration in different human tumors and clinical outcomes [11, 12]. Meningioma, an immune-sensitive malignant tumor, is infiltrated by numerous immune cells, including CD8 lymphocytes, macrophages and mast cells (MCs) [13]. In different types of meningiomas, a variety of phenotypes of MCs are found primarily in the lobule of connective tissues, including malignancies independent of growth rate, grade, or degree of calcification. In the last several decades, the importance of mast cells under several physiological and pathological conditions, has been described. However, their molecular mechanism in meningioma development remains unknown [14]. In the present study, we retrieved the meningioma-associated microarray data from Gene Expression Omnibus (GEO) databases in order to investigate tumor immune cell-related genes, transcription factors (TF), functional processes, and pathways that could be significantly correlated with meningioma development and overall patient survival. Further analysis performed included tumor-immune cell related Differentially Expressed Gene (DEG) screening, Gene Ontology (GO), Kyoto Encyclopedia of Genes and Genomes (KEGG) enrichment analysis, survival analysis, TF-miRNA- mRNA and TF-miRNA- mRNA construction, Protein-Protein Interaction (PPI), and drug-gene construction.

## Methods

### Data source

In this study, the GSE43290 dataset containing the gene expression profiles from meningioma tumor tissues obtained from 47 patients and 4 normal control samples, was downloaded from the Gene Expression Omnibus (GEO) database (GSE43290: updated August 10, 2018; https://www.ncbi.nlm.nih.gov/geo/) and accessed with GPL96 [HG-U133A] Affymetrix Human Genome U133A Array platform. GSE77259 dataset, which contained 17 samples (14 meningioma tumor tissues samples and 3 normal samples) was selected as the validation data, and assessed by the platform of [HuGene-1_0-st] Affymetrix Human Gene 1.0 ST Array. For survival analysis, the GSE16581 dataset (updated May 25, 2019) including the overall-survival (OS) data for 68 meningioma patients was accessed using the GPL570 [HG-U133_Plus_2] Affymetrix Human Genome U133 Plus 2.0 Array platform.

### Data preprocessing and identification of DEGs

According to the Series Matrix data Files of GSE43290, GSE77259 and GSE16581, each dataset was normalized independently using Robust Multiarray Average (RMA) algorithm in the Affy Bioconductor package in R [15, 16]. The Bioconductor annotation packages for GSE43290 and GSE16581 were specifically hgu133a.db, hugene10sttranscriptcluster.db and hgu133plus2.db. Afterwards, the Facto MineR package in R was used for principal component analysis (PCA) and clustering.

To identify the DEGs between meningioma and normal samples, the improved t-test method based on empirical bayes provided by the limma R package (Version: 3.40.6) [17] was used with the threshold of P. value< 0.05 and |logFC| > 1. Volcano Plot of DEGs was obtained through the ggpubr package in R (version 0.2.2), and hierarchical clustering was performed using pheatmap package in R (|logFC| > 2). Principal Component Analysis (PCA) and clustering analysis for the DEG were conducted using the FactoMineR package in R with |logFC| > 2.

### Analysis of abundance of infiltrating immune cells

The CIBERSORT deconvolution algorithm [18] was used to estimate the matrix of abundant immune cells on the expression matrix of the samples, so as to analyze the abundance of infiltrating immune cells. The parameters were set as perm = 100 and QN = TRUE. The barplot, pheatmap and vioplot were plotted using R language. *P* < 0.05 was considered as subsets of immune cells with significant differences.

### Screening immune cell related modules and genes

Based on tumor samples of immune maceration, the DEGs were analyzed by using the WGCNA package in R software (Version 1.68) [19]. The gene sets that significantly correlated with the degree of infiltration of different immune cell subsets were screened, and the genes were found to be related to the immune cell subsets. The boxplot of immune-related DEGs between tumor and normal samples was constructed based on the ggboxplot function of ggpubr package in R.

### Survival analysis

Survival analysis was performed using Survival package (version 2.44–1.1) with log rank test (cut off: P.value< 0.05) was used based on the immune-related DEGs and clinical information of GSE16581, and survival curve was constructed. Univariate and multivariate Cox regression analysis was conducted using the coxph method in survival package [20], and ggforest package in R was used for HR forest mapping construction.

### Construction of TF-miRNA- mRNA co-expression network

miRWalk3.0, miRDB, TargetScan, and miRTarBase were used to predict miRNA-mRNA interactions. TF-mRNA pairs were obtained using the online database TRRUST (https://www.grnpedia.org/trrust/) [21]. The co-expression network was constructed using Cytoscape software, and enrichment analysis was carried out for transcription factors of DEGs in the risk model.

### Functional enrichment analysis

For the functional annotation of DEGs, the ClusterProfiler package in R (Version 3.12.0) was used to identify the enriched GO terms and KEGG pathways [22]. The results were considered to be significant with FDR adjusted *p* < 0:05.

### Screening of pathways associated with meningioma

Out of the pathways associated with meningioma in CTD (http://ctdbase.org/, KEGG pathway enriched by transcription factors and immune-related genes was further screened, and a pathway diagram drawn with the pathview package in R (version 1.24.0).

### Protein-protein interaction (PPI) network construction

In order to further predict the interaction of protein pairs, the Search Tool for the Retrieval of Interacting Genes (STRING) database [23] was used with a confidence score > 0.7. And the PPI integrated networks were mapped by Cytoscape 3.4.0 software [24]. Finally, MCODE [25] was used to screen the modules of hub genes from the PPI network, with the node Score > =10 as cut-off criterion.

### The prediction of drug-gene interaction

Drug-gene interaction was predicted by Drug Gene Interaction database (DGIdb) 3.0 (Version: 3.0.2) [26], and the network was constructed with the Cytoscape software. The DrugBank database [27] was used to retrieve information about predicted drugs.

## Results

### Identification of DEGs

Following differential gene expression analysis and filtering with the cut-off criteria of P. value < 0.05 & |logFC| > 1, the DEGs between tumor and normal cells were screened. For the GSE43290 dataset, a total of 1499 DEGs were identified, out of which 73 were up-regulated and 1426 were down-regulated. For the GSE77259 dataset, 4830 DEGs, including 2536 up-regulated and 2294 down-regulated genes, were identified. Moreover, a total of 673 overlapping DEGs, such as *MYC*, *TNFAIP3* and *SLC2A3* in both GSE43290 dataset and GSE77259 dataset were obtained (Supplementary Fig. 1).

**Fig. 1:**
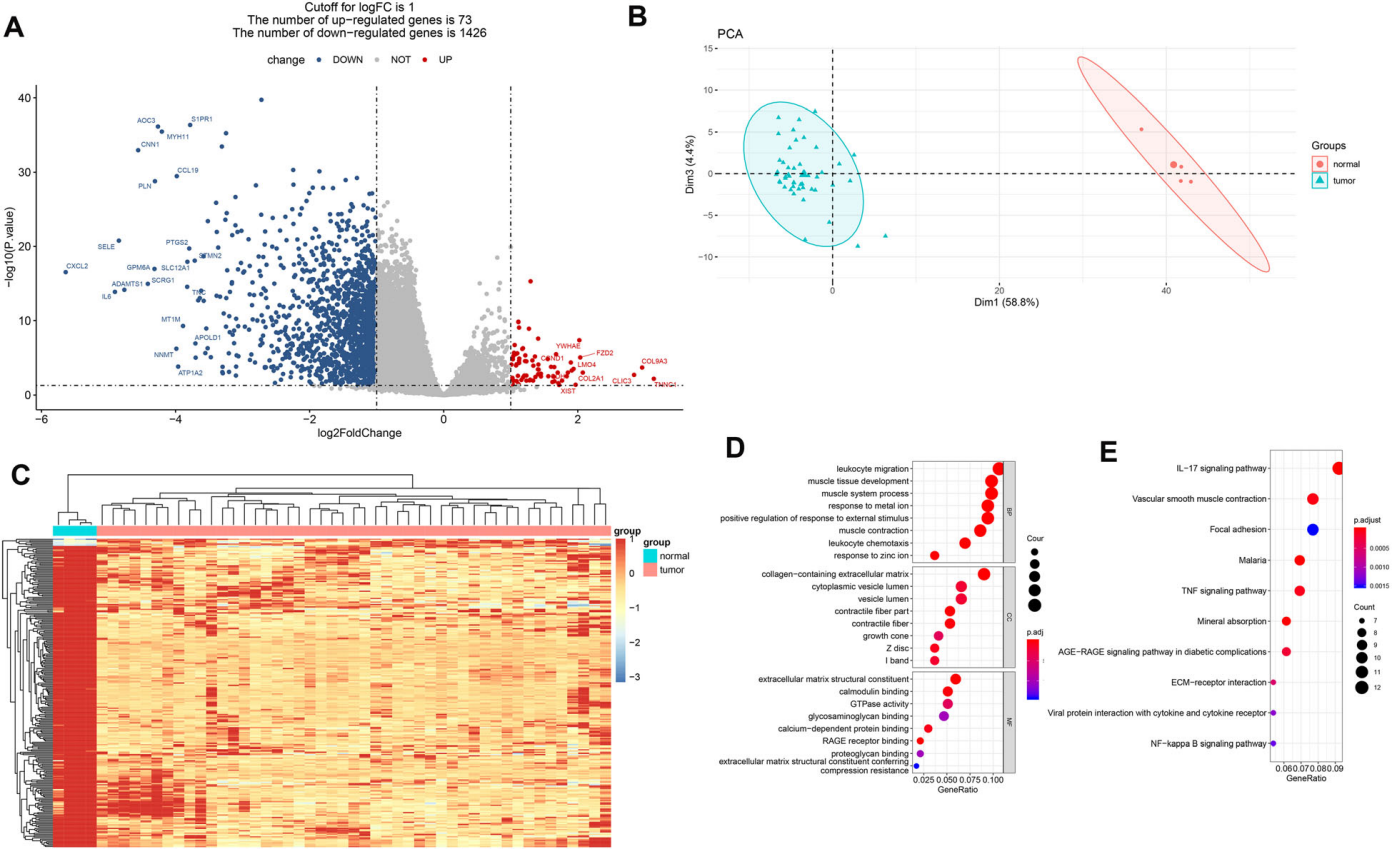
Differential expression analysis and function enrichment. (**A**), volcano plot of differentially expressed genes. The blue dots represent down-regulated genes, red dots represent up-regulated genes and grey dots represent the genes without significant expression changes between meningiomas and normal gorups; (**B**), the principal component analysis of all samples shows the significant differences between these two groups; (**C**), heatmaps of differentially expressed genes. The light blue and pink blocks in the top represent normal and meningiomas samples, respectively. The Y-axis represent all the differentially expressed genes; Bubble diagrams show the significantly enriched gene ontology annotation terms (**D**) and KEGG pathways (**E**). The size of bubbles represent the count of genes, and thre color from red to blue represent the *P* value from samll to large

Furthermore, volcano plot and PCA clustering of DEGs in GSE43290 showed that the DEGs between the two groups could be significantly distinguished (Fig. 1A and B). Additionally, using a threshold of |logFC| > 2, 255 DEGs out of the 1499 DEGs in GSE43290 were further screened, and the expression pattern of these DEGs could divid the samples into two groups (Fig. 1C). To further predict the potential functions of these DEGs in meningioma, enrichment analysis was performed. The results of GO analysis showed that these DEGs were functionally classified by Biological Process (BP), Cellular Component (CC), and Molecular Function (MF) (Fig. 1D). Among them, several BPs related to immune response (leukocyte migration, leukocyte chemotaxis) and responses processes (reponse to metal ion, response to zinc ion, positive regulation of response to external stimulus) were significantly enriched; moreover, CCs and MFs were found to be mainly associated with regulation of cardiac electrical activity. Furthermore, KEGG pathway analysis revealed a series of enriched pathways, such as IL-17 signaling pathway and TNF signaling pathway (Fig. 1E).

### The immune cell infiltration landscape

**Fig. 2:**
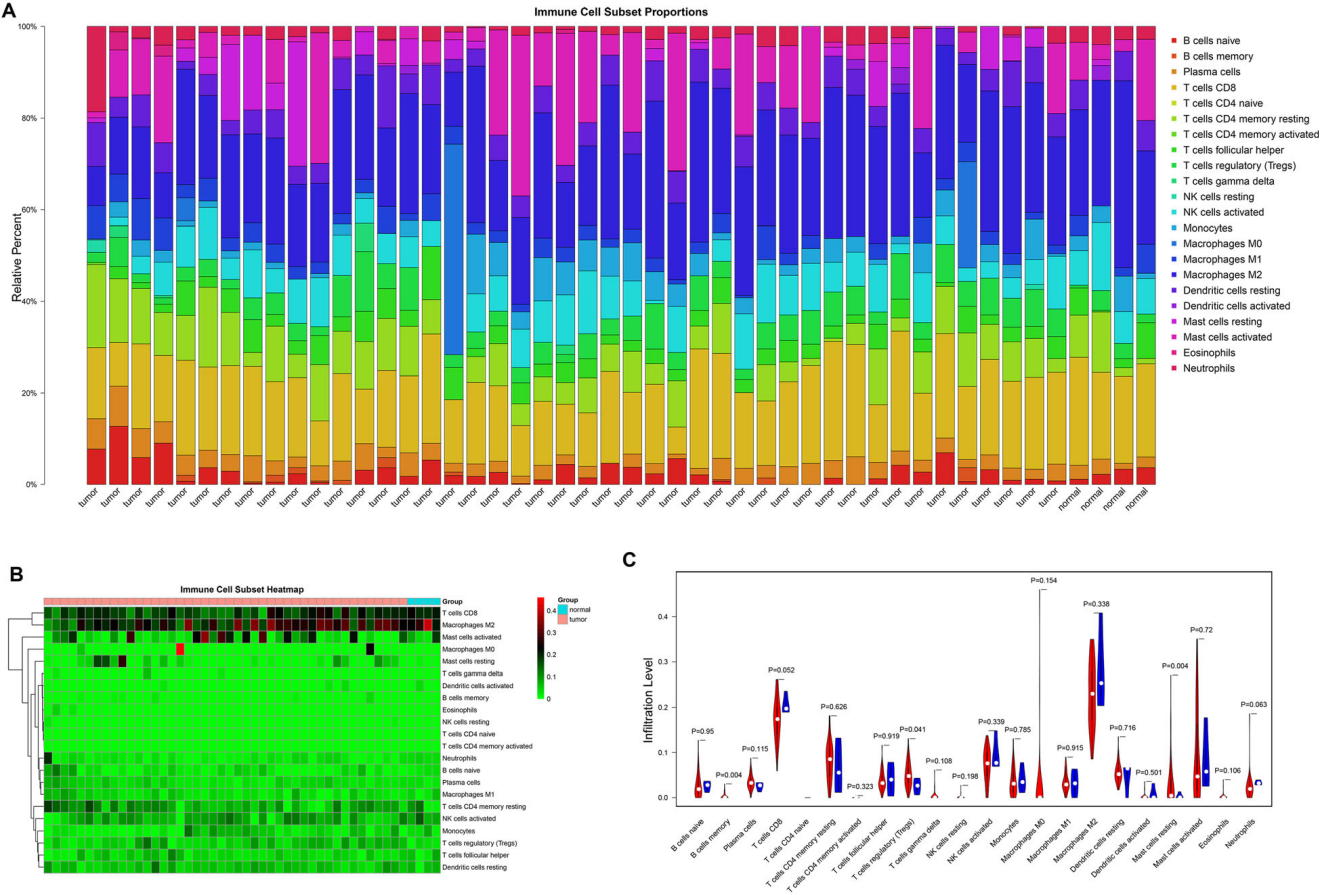
The landscape of immune cells infiltration in meningiomas cohort. (**A**) Histogram show the landscape of 22 immune cells infiltration in all samples. X-axis represent samples and Y-axis represent the proportions of each immune cell infiltration; (**B**) Heatmap of the 22 immune cell proportions. X-axis represent samples and Y-axis represent the infiltration abundance of 22 immune cells. Color from red to green represent the infiltration abundance from high to low; (**C**) The violin plot show the difference on infiltration abundance of 22 immune cells between meningiomas and normal samples. Red represents tumor group, and blue represents the normal group

Based on the gene expression profiles of GSE43290, the difference in immune cells infiltration between tumor and control samples among 22 immune cells types, were detected using CIBERSORT algorithm. As shown in Fig. 2A, 48 of the 51 samples were valid (*P* value < 0.05), including 44 tumor samples and 4 normal samples. Among the 22 immune cell types, the percentage of CD8+ T cells (yellow) and M2 macrophages (blue) in each sample was relatively high. The heatmap showing the 22 tumor immune cells was illustrated in Fig. 2B, which also confirmed the high infiltrating abundance of CD8+ T cells and M2 macrophages in all samples. Based on the violin plot (Fig. 2C), the probability of distribution of memory B cells, regulatory T cells (Tregs) and resting mast cells in tumor were obviously higher than those in the control samples (all, *P* < 0.05), indicating that the immune infiltration proportions could be useful in distinguishing meningioma patients from healthy patients.

In addition, a lot of literature have shown that mast cells (MC) were associated with the development of meningioma [13, 14, 28], and resting mass cells infiltration ratio was relatively high in this study. Hence, the follow-up analysis focused on resting mast cells.

### Weighted correlation network analysis (WGCNA) co-expression networks

When correlation coefficient threshold was set at 0.85, the soft-thresholding power was 6 (Supplementary Fig. 2A). Through WGCNA analysis, 10 co-expression modules were constructed based on the 1499 DEGs (Supplementary Fig. 2B), of which the grey module was the gene set that could not gather into other modules, so there were only 9 valid gene modules.

**Fig. 3:**
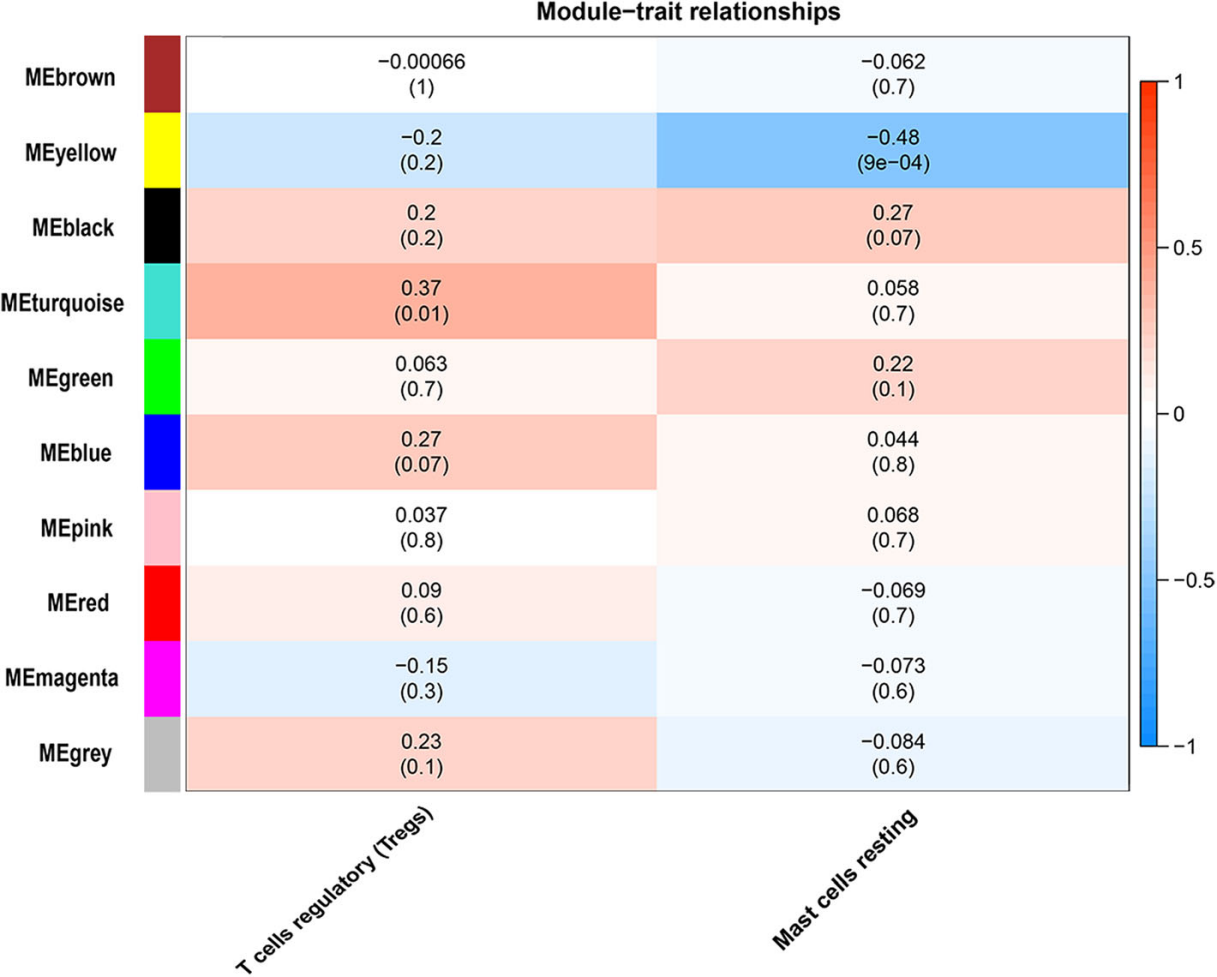
The module-trait relationships in WGCNA analysis. The plot show the correlations of gene modules with the infiltration abundance of regulatory T cells (Tregs) and resting mast cells. The number in each block represent correlation coefficient and *P* value (numbers in parentheses), respectively

Subsequently, as shown in Fig. 3, the correlation between each module and the degree of invasion of immune cells in tumor tissue was calculated. The results revealed that yellow, the module with the highest correlation, contained 158 DEGs, consisting of 2 up-regulated and 156 down-regulated genes (Supplementary Table 1). Yellow modules are negatively related to degree of resting mast cells infiltration in meningioma tissues, and the correlation between genes in the yellow module and the degree of immune cell infiltration was summarized in the Supplementary Table 2.

**Fig. 4:**
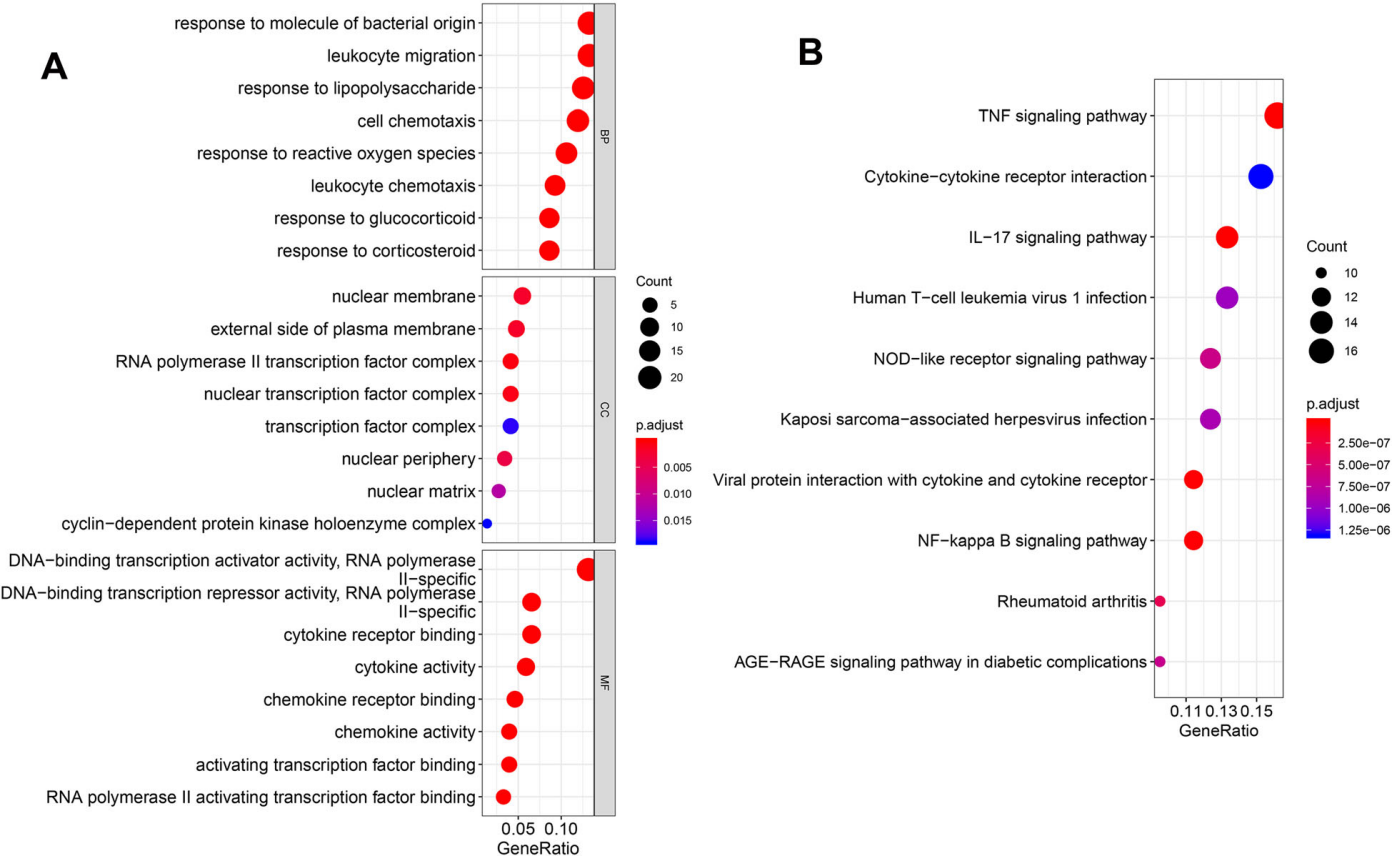
Functional analysis of differential expression genes related to resting mast cells infiltration. Bubble diagrams show the significantly enriched gene ontology annotation terms (**A**) and KEGG pathways (**B**). The size of bubbles represent the count of genes, and thre color from red to blue represent the *P* value from samll to larger. The BP, CC and MF in gene ontology annotation terms are biological processes, cellular component and molecular function, respectively

Furthermore, GO functional analysis revealed that the genes in yellow module were dominant in biological processes involved in response to leukocyte migration, molecules of bacterial origin, and inflammatory response (Fig. 4A); KEGG pathway analysis revealed that the genes were mainly enriched in the TNF signaling pathway, cytokine-cytokine receptor interaction and IL-17 signaling pathway (Fig. 4B).

### Risk model construction of key genes

**Fig. 5:**
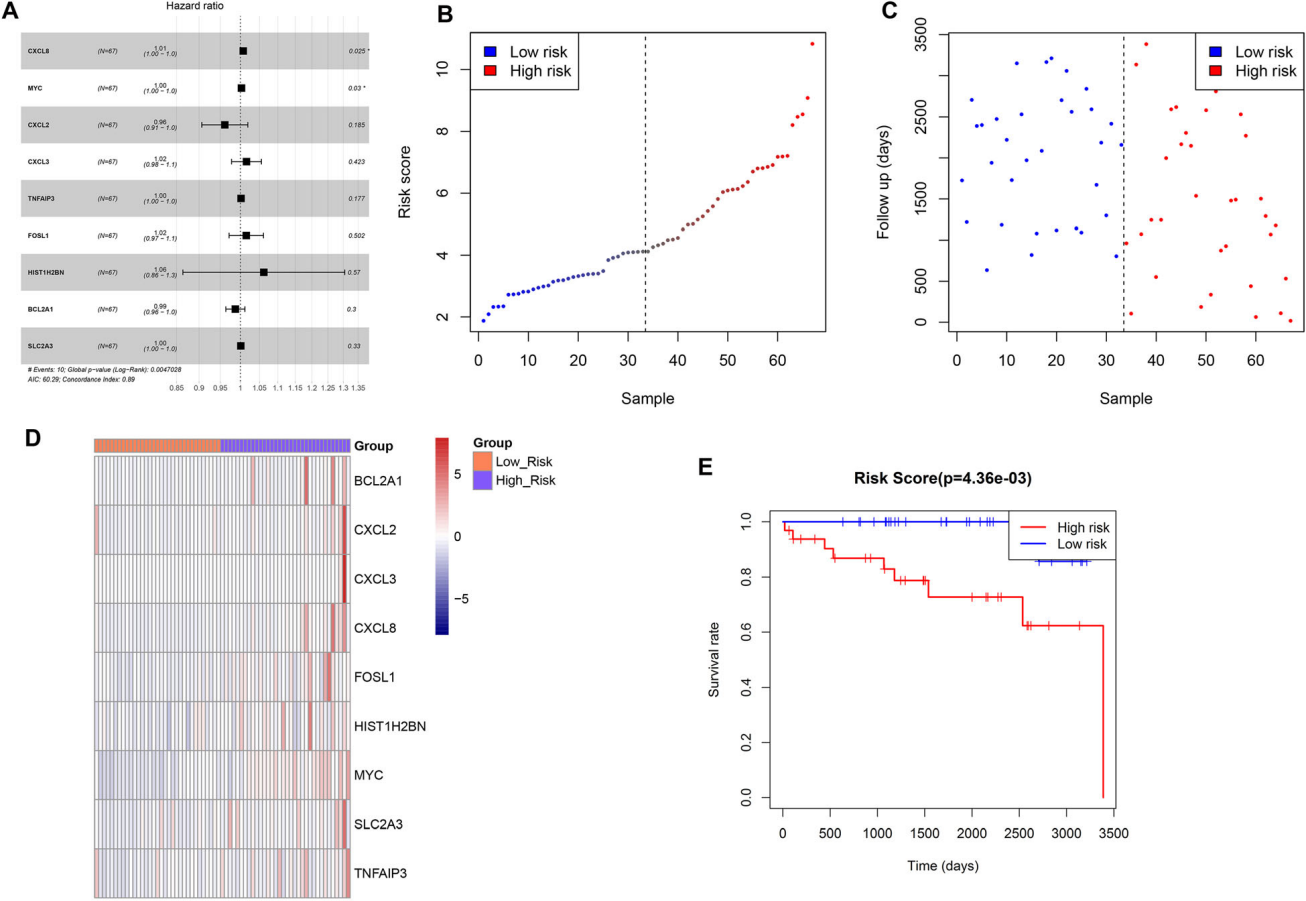
Construction of prognostic risk model. (**A**) Forest plot show the hazard ratio of the nine prognosis-related genes in Cox multivariate analysis; (**B**) The classification curve of the sample risk model; (**C**) Survival time scatter plot of risk model; (**D**) heatmap show the expression pattern of nine genes in risk model with the risk score from low to high. The color from red to blue represent genes expression from high to low; (**E**) The K-M survival curveshow the difference on overall survival of patients in high risk and low risk groups

In order to identify whether each key gene related to resting mast cells correlated with prognosis of meningioma patients, univariate and multivariate analyses were performed. K-M survival analysis revealed that *CXCL8* and *MYC* were associated with prognosis of meningioma patients. Moreover, based on the COX univariate and multivariate regression analyses showed *CXCL8* and *MYC* might be prognostic biomarkers for meningioma patients (Table 1 and Fig. 5A).

**Table 1:** The univariate and multivariate Cox regression analysis

Gene	Univariate analysis				Multivariate analysis			
	HR	lower.95	upper.95	*p*.val	HR	lower.95	upper.95	*p*.val
CXCL8	1.002	1.001	1.004	0.001	1.007	1.001	1.014	0.025
MYC	1.002	1.001	1.004	0.003	1.003	1.000	1.005	0.030
CXCL2	1.010	1.003	1.017	0.006				
CXCL3	1.007	1.002	1.013	0.008				
TNFAIP3	1.002	1.001	1.004	0.010				
FOSL1	1.031	1.002	1.061	0.036				
HIST1H2BN	1.147	1.008	1.305	0.038				
BCL2A1	1.007	1.000	1.014	0.038				
SLC2A3	1.001	1.00	1.002	0.0495

Subsequently, based on the 9 genes in univariate regression analysis above, the risk model was constructed. Firstly, the meningioma patients were categorized into low- or high-risk patients, and the cut off value was the median risk score (Fig. 5B). The scatter plot of survival time of risk model samples showed that the survival time of samples was relatively lower in the high-risk group the low-risk group (Fig. 5C). Monolayer clustering analysis of RNA expression in the risk model of each sample showed differences in the key genes between high and low risk groups (Fig. 5D). Moreover, K-M survival curve indicated that the survival time between the high and low risk groups was significantly different (*P* value = 0.00436; Fig. 5E), suggesting the risk model of 9 key gene related to resting mast cells was successfully constructed.

### TF-miRNA- mRNA co-regulation network

**Fig. 6:**
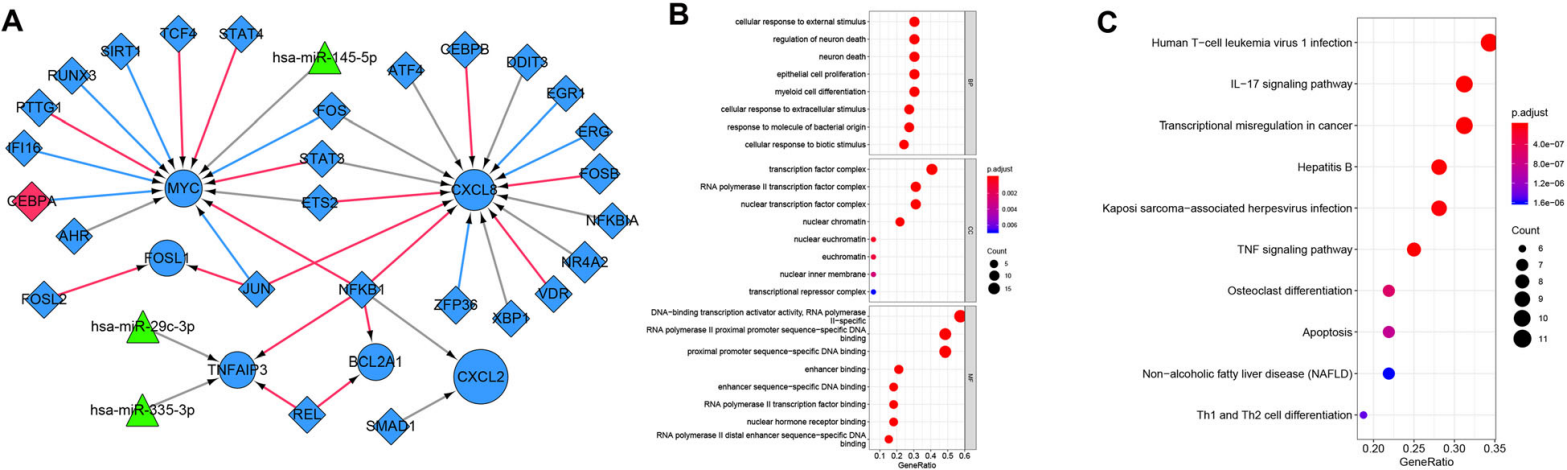
Regulatory network for the genes in prognostic risk model. (**A**) The miRNA-transcription factor (TF)-mRNA regulatory network. The circle nodes represent the genes in prognostic risk model, rhombus nodes represent the predicted TFs, and triangles represent the predicted miRNAs. Bule and red nodes represent down-regulated and up-regulated, respectively. Bubble diagrams show the significantly enriched gene ontology annotation terms (**B**) and KEGG pathways (**C**) for the genes and TFs in the regulatory network. The size of bubbles represent the count of genes, and thre color from red to blue represent the *P* value from samll to larger. The BP, CC and MF in gene ontology annotation terms are biological processes, cellular component and molecular function, respectively

Based on the database (miRWalk3.0, TargetScan, MiRDB, and MirTarBase) with the Score > 0.95, 145 miRNAs acting on 3’UTR region of the genes in the risk model associated with resting mast cells were predicted. Then, according to the retrieval of HMDD V3.2 database, 3 miRNA-mRNA pairs (including MYC-miR-145-5p, TNFAIP3-miR-29c-3p and TNFAIP3-miR-335-3p) were obtained, respectively. Subsequently, and 37 TF-mRNA pairs (including 6 mRNAs and 27 TFs) were screened using the online database of TRRUST. Lastly, based on mRNA-miRNA pairs and TF-mRNA pairs, miRNA-TF-mRNA co-regulation network was constructed using Cytoscape (Fig. 6A). The nine genes in prognostic risk model and the TFs that regulated their expression were considered as important genes in meningeoma. Therefore, expression of these genes and TFs were verified using an external dataset GSE54934. Except for HIST1H2BN, the expression of all genes in prognostic risk model showed decreased trend in meningeoma compared to normal, especially in the expression of CXCL2 and SLC2A3 (Supplementary Fig. 3A). In addition, the expression of the 27 TFs showed decreased trend in meningeoma than normal, especially in the expression of CEBPB, DDIT3, ETS2, FOSL2, SMAD1, TCF4. These findings were consistent with our results (Supplementary Fig. 3B). However, not all genes showed significant differences, and this might be explained by the difference in sample size in meningeoma (*n* = 22) and normal groups (*n* = 3).

Additionally, in order to further investigate the potential biological functions of the genes involved in miRNA-TF-mRNA co-regulation network, the GO terms and KEGG pathway analyses were conducted. As illustrated in Fig. 6B, the functional processes of the genes were primarily related to biological regulation, while KEGG pathway analysis revealed that the genes were enriched in Tumor Necrosis Factor (TNF) signaling pathway and IL-17 signaling pathway (Fig. 6C).

### PPI network and modules analysis

**Fig. 7:**
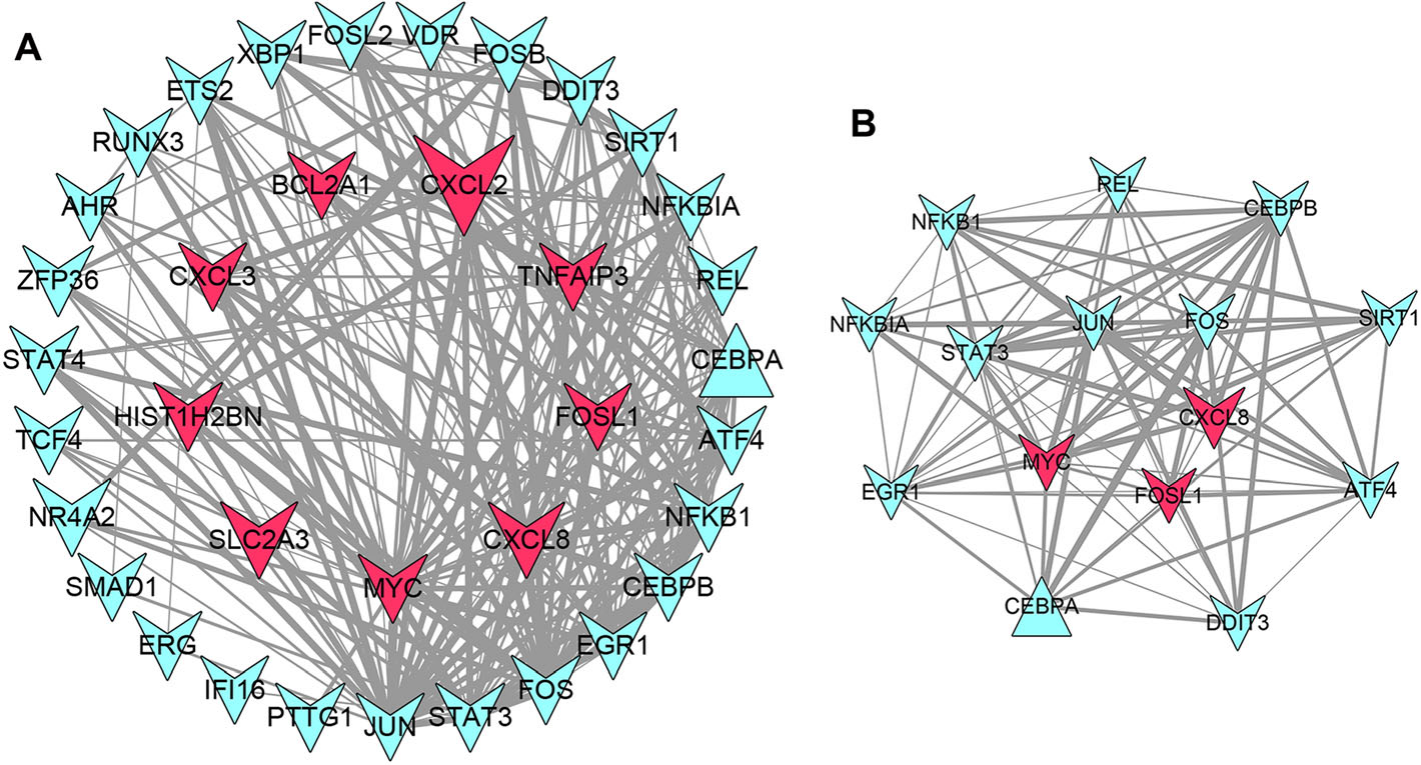
The protein-protein interaction (PPI) network and modules analysis. (**A**) The PPI network for the nine genes in prognostic risk model and the predicted TFs in the regulatory network. (**B**) The significant module with score > 12 identified from the PPI network. The triangle node represents up-regulated differential gene; Arrowhead nodes represent down-regulated differential genes; The red nodes represent the differential genes in the prognostic model, and the blue nodes represent other differential genes (TFs in the regulatory network)

According to STRING database, the PPI network (PPI score = 0.4) was constructed with 27 TFs in miRNA-TF-mRNA co-regulation network and 9 DEGs in the risk model using Cytoscape. As shown in Fig. 7A, there were 36 nodes and 197 interactions in the PPI network. With score > 12, a module with 15 nodes and 88 interactions was further revealed from the PPI network using the MCODE of Cytoscape software (Fig. 7B).

### Drug-gene network

**Fig. 8:**
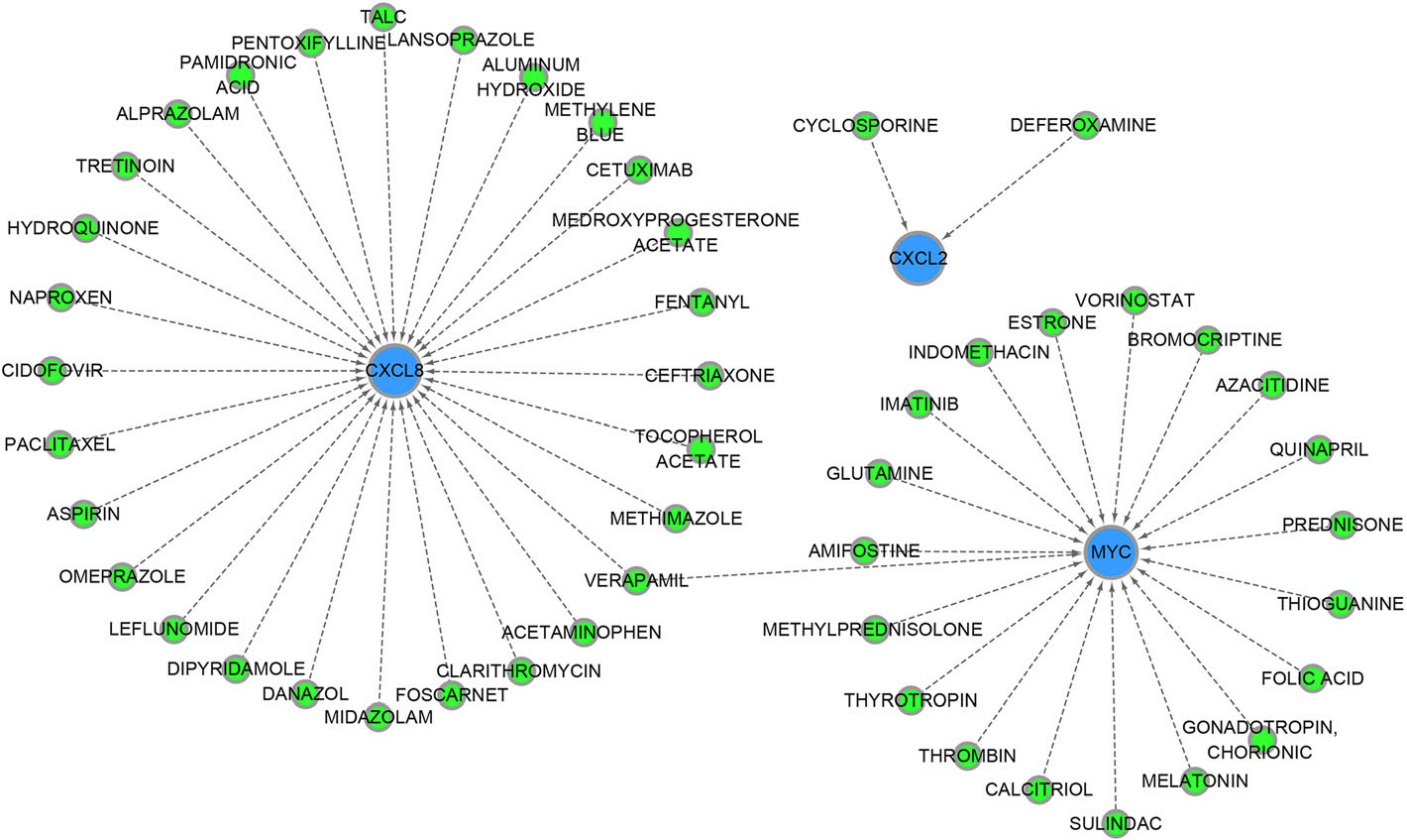
Drug-gene network for the genes in prognostic risk model. The network shows the predicted targeted interactions between small molecule drugs and the genes in prognostic risk model. Blue represents downregulated genes in prognostic risk model, and green represents drugs, respectively. The lines represent there is targeted interaction between small molecule drug and gene

Similarly, based on the 9 DEGs in the risk model, a total of 50 drug-gene interacting pairs, including 3 target genes (*CXCL2*, *CXCL8* and *MYC*), and 49 drugs were identified using the DGIdb 3.0 database. Furthermore, drug-gene interaction network was constructed by Cytoscape software (Fig. 8).

## Discussion

Till now, the etiology of meningioma has not been fully elucidated, however, it is believed to involve environmental and genetic factors, with genetic factors being the important factors determining its development. Hence, in the present study, according to the microarray data from GEO database, we screened a series of genes, including *MYC* and *CXCL8*, as well as the TNF signaling pathway, cytokine-cytokine receptor interaction, and IL-17 signaling pathway, related to resting mast cells which are involved meningioma pathogenesis and prognosis. Moreover, based on the 9 key genes related to resting mast cells in meningioma, the PPI network, TF-miRNA- mRNA network, drug-gene interaction network were constructed, respectively; among which, the key miRNAs and TF that might play important roles in meningioma were selected.

Tumor immunotherapy has achieved promising results in clinical application [29, 30]. To identify new indicators for meningioma prognosis improvement, an increasing number of reports have focused on tumor-infiltrating immune cells, in accordance with the function and composition of tumor cells in cancer occurrence and development [13, 14, 31–33]. In our study, based on the analysis of abundant infiltrating immune cells (CIBERSORT deconvolution algorithm with the parameters of perm = 100 and QN = TRUE), we found that the probability of distribution of memory B cells, regulatory T cells (Tregs) and resting mast cells in tumor samples was significantly higher than that in control samples. Actually, although some literature showed that T cells regulatory cells (Tregs) associated with Meningioma [30–32], our subsequent analysis of T cell regulation did not turn out well. In addition, there is increasing underlying evidence of the association between mast cells and meningioma development [13, 14, 34], but the molecular mechanism of mast cells in meningioma immunotherapy remains unclear.

In order to explore the genes related to infiltration of resting mast cells in meningioma tissue, WGCNA analysis with R language package was performed on DEGs of meningioma patients and normal controls. The results showed yellow modules are negatively related to the degree of infiltration of resting mast cells in meningioma tissues, thus, genes such as *MYC*, *CXCL2*, *CXCL8* and *FOSL1* in the module, are inversely associated with the degree of mast cell infiltration. Pathway analysis revealed that the genes were mainly enriched in the TNF signaling pathway, cytokine-cytokine receptor interaction, and IL-17 signaling pathway. It has been reported that the cytokine-cytokine receptor interaction signaling pathway is involved in the meningioma. Additionally, although the TNF signaling pathway or IL-17 signaling pathway is rarely studied in meningioma, many researches have revealed the activation of the TNF or IL-17 signaling pathway in various brain diseases such as neuroinflammatory injury [35], autoimmune encephalomyelitis [36], and ischemic stroke [37]. Moreover, survival analysis (Survival package with log-rank test; threshold value of *P* value < 0.05) of key genes related to resting mast cells showed that the risk model constructed based on 9 key genes (*CXCL8*, *MYC*, *CXCL2*, *CXCL3*, *TNFAIP3*, *FOSL1*, *HIST1H2BN*, *BCL2A1* and *SLC2A3*) could predict the prognosis of patients with meningioma. A previous study reported that *MYC* expression is dysregulated in human meningioma, indicating its potential role in oncogenic processes [38]. Cai et al. [39] found that c-MYC in meningioma is targeted by RIZ1 to negatively regulate the ubiquitin-binding enzyme E2C/UbcH1. It has also been found that the methylation of Werner syndrome protein is associated with invasive meningioma occurrence and development via *MYC* expression regulation [40]. More importantly, *MYC* is associated with the TNF signaling and cytokine-cytokine receptor interaction pathways. Similarly, it has been found that CXC receptor activates ERK1/2 and stimulates meningioma cell proliferation [41], and in systematic investigation of quercetin for cardiovascular disease treatment, CXCL8 is enriched in the TNF signaling and IL-17 signaling pathways [42]. Hence, we speculate that the key genes, including *MYC* and *CXCL8*, are involved in meningioma progression via the regulating of different pathways such as the TNF signaling pathway, cytokine-cytokine receptor interaction, and IL-17 signaling pathway.

With the increasing studies on miRNAs, researchers have reported that miRNAs are involved in the development of several cancer types [43]. In the present study, based on the genes in the prognostic model associated with resting mast cells, 3 miRNA, including miR-145-5p, miR-29c-3p, and miR-335-3p, were predicted based on the databases (miRWalk3.0, TargetScan, MiRDB, and MirTarBase) with the Score > 0.95, and the miRNAs-TFs-mRNA co-regulation network was constructed. Among these target miRNAs, miR-29c-3p is reportedly down-regulated in meningioma [44], and Dalan et al. [45] indicated that low expression of miR-29c-3p correlated significantly with higher recurrence rates in meningioma patients. However, no evidence of a correlation between hsa-miR-145-5p, or miR-335-3p and meningioma was reported till now. Notwithstanding, miR-145-5p is linked to psychiatric and neurodegenerative disorders [46]. Further, circPTN can sponge miR-145-5p to promote stemness or proliferation in glioma [47]. Hence, based on the findings, we speculate that the downregulation of these miRNAs, including miR-29c-3p and miR-145-5p associated with resting mast cells may cause meningioma.

There were also some limitations in this study. Microarray studies of meningioma are limited owing to a lack of human disease tissues or appropriate disease models. There were obvious difference on the sample size in meningioma and normal groups, which might be an influencing factor for results. In addition, the results should be confirmed by clinical samples and data, including the infiltrating abundance of mast cells, the expression of genes in prognostic model, and the prognpstic value of the risk model. Moreover, further functional assays were lacked to confirm the regulatory mechanism in TF-miRNA-mRNA network and the proposed hypothesis.

In conclusion, this study conducted a bioinformatics analysis of DEGs related to resting mast cells, and deregulated pathways based on GSE43290, GSE77259 and GSE16581 datasets. DEGs of *MYC* and *CXCL8*, miRNAs of miR-29c-3p and miR-145-5p, as well as pathways such as the TNF signaling pathway, cytokine-cytokine receptor interaction, and IL-17 signaling pathway, probably involved in meningioma development, were obtained. These findings could improve the understanding of the pathogenesis and molecular mechanisms of resting mast cells in meningioma. Taken together, this was the first study to explore gene signatures related to resting mast cells in meningioma by a bioinformatics analysis. Moreover, this study combined immune infiltration with prognostic risk models in the meningioma direction; meanwhile, CIBERSORT deconvolution algorithm was used to quantify the infiltration abundance of each immune cell, and the correlation between modules and immune cells was calculated. However, further clinical studies are required to confirm the function of the identified genes.

## Supplementary Information

**Additional file 1: Supplementary Table 1.** The differentially expressed genes in yellow module.

**Additional file 2: Supplementary Table 2.** The correlation between genes in the yellow module and the degree of immune cell infiltration.

**Additional file 3: Supplementary Fig. 1.** Venn plot shows the common genes in the two datasets

**Additional file 4: Supplementary Fig. 2.** WGCNA revealed gene co-expression networks. (A) Analysis of the scale-free fit index for various soft thresholding powers (Left) and analysis of the mean connectivity for various soft-thresholding powers (Right). (B) Network heatmap plot in the co-expression modules.

**Additional file 5: Supplementary Fig. 3.** Expression of genes verified by an external dataset GSE54934. (A) Boxplot shows the expression of the nine genes in prognostic risk model between meningeoma and normal samples; (B) Boxplot shows the expression of the 27 transcription factors between meningeoma and normal samples.

## Data Availability

All data generated or analyzed during this study are included in this published article.

## References

[1] Ostrom QT, Gittleman H, Farah P, Ondracek A, Chen Y, Wolinsky Y, et al. CBTRUS Statistical Report: Primary Brain and Central Nervous System Tumors Diagnosed in the United States in 2006–2010. Neuro-Oncology. 2013;15(suppl 2):ii1–ii56. 10.1093/neuonc/not151. 10.1093/neuonc/not151 PMC3798196 24137015

[2] Rogers L, Barani I, Chamberlain M, Kaley TJ, Mcdermott M, Raizer J, et al. Meningiomas: knowledge base, treatment outcomes, and uncertainties. A RANO review. J Neurosurg. 2015;122(1):4–23. 10.3171/2014.7.JNS131644. 10.3171/2014.7.JNS131644 PMC5062955 25343186

[3] Maxwell M, Galanopoulos T, Neville-Golden J, Antoniades HN (1993). Expression of androgen and progesterone receptors in primary human meningiomas. J Neurosurg..

[4] Probst-Cousin S, Villagran-Lillo R, Lahl R, Bergmann M, Schmid KW, Gullotta F (1997). Secretory meningioma: Clinical, histologic, and immunohistochemical findings in 31 cases. Cancer..

[5] Madhusoodanan S, Patel S, Reinharth J, Hines A, Serper M (2015). Meningioma and psychiatric symptoms: a case report and brief review. Ann Clin Psychiatry.

[6] El-Zein R, Bondy M, Wrensch M (2005). Epidemiology of brain tumors.

[7] Nakasu S, Fukami T, Jito J, Nozaki K (2009). Recurrence and regrowth of benign meningiomas. Brain Tumor Pathol..

[8] Xiong YF, Wang K, Zhou H, Peng LL, You WX, Fu ZX (2018). Profiles of immune infiltration in colorectal cancer and their clinical significant: a gene expression-based study. Cancer Med..

[9] Liu X, Wu S, Yang Y, Min Z, Hou Z (2017). The prognostic landscape of tumor-infiltrating immune cell and immunomodulators in lung cancer. Biomed Pharmacother.

[10] Mony JT, Schuchert MJ (2018). Prognostic Implications of Heterogeneity in Intra-tumoral Immune Composition for Recurrence in Early Stage Lung Cancer. Front Immunol..

[11] Pagès F, Galon J, Dieu-Nosjean M-C, Tartour E, Sautès-Fridman C, Fridman W-H (2010). Immune infiltration in human tumors: a prognostic factor that should not be ignored..

[12] Viel S, Charrier E, Marçais A, Rouzaire P, Bienvenu J, Karlin L, et al. Monitoring NK cell activity in patients with hematological malignancies. Oncoimmunology. 2013; 2(9):e26011. 10.4161/onci.26011. 10.4161/onci.26011 PMC3850490 24327939

[13] Polyzoidis S, Koletsa T, Panagiotidou S, Ashkan K, Theoharides TC (2015). Mast cells in meningiomas and brain inflammation. J Neuroinflammation..

[14] Tirakotai W, Mennel HD, Celik I, Hellwig D, Bertalanffy H, Riegel T (2006). Secretory meningioma: immunohistochemical findings and evaluation of mast cell infiltration. Neurosurg Rev..

[15] Gentleman RC, Carey VJ, Bates DM, Bolstad B, Dettling M, Dudoit S. Bioconductor: open software development for computational biology and bioinformatics. Genome Biol. 2004;5(10):R80. 10.1186/gb-2004-5-10-r80. 10.1186/gb-2004-5-10-r80 PMC545600 15461798

[16] Gautier L, Cope L, Bolstad BM. Irizarry RA. affy—analysis of Affymetrix GeneChip data at the probe level. Bioinformatics. 2004;20(3):307–15. 10.1093/bioinformatics/btg405. 10.1093/bioinformatics/btg405 14960456

[17] Smyth GK (2011). limma: Linear Models for Microarray Data.

[18] Newman AM, Liu CL, Green MR, Gentles AJ, Feng WG, Xu Y (2015). Robust enumeration of cell subsets from tissue expression profiles. Nat Methods..

[19] Langfelder P, Horvath S (2008). WGCNA: an R package for weighted correlation network analysis. BMC Bioinformatics..

[20] COX D (1972). Regression models and life tables. J R Stat Soc.

[21] Han H, Jae-Won C, Sangyoung L, Yun A, Hyojin K, Dasom B, Yang S, Yeong KC, Muyoung L, Eunbeen K (2017). TRRUST v2: an expanded reference database of human and mouse transcriptional regulatory interactions. Nucleic Acids Res.

[22] Kanehisa M, Goto S (2000). KEGG: Kyoto encyclopedia of genes and genomes. Nucleic Acids Res.

[23] Szklarczyk D, Morris JH, Cook H, Kuhn M, Wyder S, Simonovic M, et al. The STRING database in 2017: Quality-controlled protein–protein association networks, made broadly accessible. Nucleic Acids Res. 45(D1):D362–8. 10.1093/nar/gkw937. 10.1093/nar/gkw937 PMC5210637 27924014

[24] Tang Y, Li M, Wang J, Pan Y, Wu FX (2015). CytoNCA: A cytoscape plugin for centrality analysis and evaluation of protein interaction networks. Biosystems..

[25] Bader GD, Hogue CW (2003). An automated method for finding molecular complexes in large protein interaction networks. BMC Bioinformatics..

[26] Cotto KC, Wagner AH, Yang-Yang F, Susanna K, Coffman AC, Gregory S, Alex W, Spies NC, Griffith OL, Malachi G (2017). DGIdb 3.0: a redesign and expansion of the drug–gene interaction database. Nucleic Acids Res.

[27] Vivian L, Craig K, Yannick D, Tim J, Guo AC, Liu Y, Adam M, David A, Michael W, Vanessa N (2013). DrugBank 4.0: shedding new light on drug metabolism. Nucleic Acids Res.

[28] Reszec J, Hermanowicz A, Rutkowski R, Bernaczyk P, Mariak Z, Chyczewski L (2013). Evaluation of mast cells and hypoxia inducible factor-1 expression in meningiomas of various grades in correlation with peritumoral brain edema. J Neurooncol..

[29] Sachpekidis C, Larribere L, Pan L, Haberkorn U, Dimitrakopoulou-Strauss A, Hassel JC (2015). Predictive value of early 18F-FDG PET/CT studies for treatment response evaluation to ipilimumab in metastatic melanoma: preliminary results of an ongoing study. Eur J Nucl Med Mol Imaging..

[30] Butterfield LH (2007). Recent advances in immunotherapy for hepatocellular cancer. Swiss Med Wkly.

[31] Domingues PH, Teodósio C, Ortiz J, Sousa P, Otero Á, Maillo A (2012). Immunophenotypic Identification and Characterization of Tumor Cells and Infiltrating Cell Populations in Meningiomas. Am J Pathol..

[32] Herold-Mende C, Ull T, Rapp C, Dettling S, Simon M. IMPS-14 Prognostic role of regulatory T-cells in primary and recurrent meningioma. Neuro-Oncology. 2015;17(suppl 5):v111–v116. 10.1093/neuonc/nov217.14.

[33] Gelerstein E, Berger A, Jonas-Kimchi T, Strauss I, Kanner AA, Blumenthal DT (2017). Regression of intracranial meningioma following treatment with nivolumab: Case report and review of the literature. J Clin Neurosci..

[34] Dai J, Ma Y, Chu SH, Le NY, Cao J, Wang Y (2018). Identification of key genes and pathways in meningioma by bioinformatics analysis. Oncol Lett..

[35] Liu DL, Zhao LX, Zhang S, Du JR (2016). Peroxiredoxin 1-mediated activation of TLR4/NF-κB pathway contributes to neuroinflammatory injury in intracerebral hemorrhage. Int Immunopharmacol..

[36] Souza PS, GonçAlves ED, Pedroso GS, Farias HR, Junqueira SC, Marcon R (2017). Physical exercise attenuates experimental autoimmune encephalomyelitis by inhibiting peripheral immune response and blood-brain barrier disruption. Mol Neurobiol..

[37] Gelderblom M, Weymar A, Bernreuther C, Velden J, Magnus T (2012). Neutralization of the IL-17 axis diminishes neutrophil invasion and protects from ischemic stroke. Blood.

[38] Jennifer K, Grenier PAF, Sloma EA, Miller AD. RNA-seq transcriptome analysis of formalin fixed, paraffin-embedded canine meningioma. PLoS One. 2017;12(10). 10.1371/journal.pone.0187150. 10.1371/journal.pone.0187150 PMC5658167 29073243

[39] Cai Z, Zou Y, Hu H, Lu C, Hu G (2017). RIZ1 negatively regulates ubiquitin-conjugating enzyme E2C/UbcH10 via targeting c-Myc in meningioma. Am J Transl Res.

[40] Li P, Hao S, Bi ZY, Zhang JT, Wu Z, Ren XH (2015). Methylation of Werner syndrome protein is associated with the occurrence and development of invasive meningioma via the regulation of Myc and p53 expression. Exp Ther Med..

[41] Barbieri F, Bajetto A, Porcile C, Pattarozzi A, Massa A, Lunardi G (2006). CXC Receptor and Chemokine Expression in Human Meningioma: SDF1/CXCR4 Signaling Activates ERK1/2 and Stimulates Meningioma Cell Proliferation. Ann N Y Acad Sci..

[42] Wu X-J, Zhou X-B, Chen C, Mao W (2019). Systematic investigation of quercetin for treating cardiovascular disease based on network pharmacology. Comb Chem High Throughput Screen.

[43] Klinge CM. Noncoding RNAs: long non-coding RNAs and microRNAs in endocrine-related cancers. Endocr Relat Cancer. 2018;25(4):R259–R282. 10.1530/ERC-17-0548. 10.1530/ERC-17-0548 29440232

[44] Galania V, Lamprib E, Varouktsic A, Alexioud G, Mitseloue A (2017). Genetic and epigenetic alterations in meningiomas. Clin Neurol Neurosurg.

[45] Zhi F, Zhou G, Wang S, Shi Y, Yang Y (2013). A microRNA expression signature predicts meningioma recurrence. Int J Cancer.

[46] HA W, D K, S W, H S, SL S, C B, M S-M, DS D, DS P, H L (2020). MicroRNA sequencing of rat hippocampus and human biofluids identifies acute, chronic, focal and diffuse traumatic brain injuries. Sci Rep.

[47] J C, T C, Y Z, Y L, Y Z, Y W, X L, X X, J W, M H (2019). circPTN sponges miR-145-5p/miR-330-5p to promote proliferation and stemness in glioma. J Exp Clin Cancer Res.

