# Supplementary figures and images for: Identification of key genes and pathways associated with resting mast cells in meningioma
Source: BMC Cancer. 2021 Nov 12;21:1209. doi: 10.1186/s12885-021-08931-0 (PMC8590208; doi:10.1186/s12885-021-08931-0)

**Supplementary figure 1** Venn plot shows the common genes in the two datasets


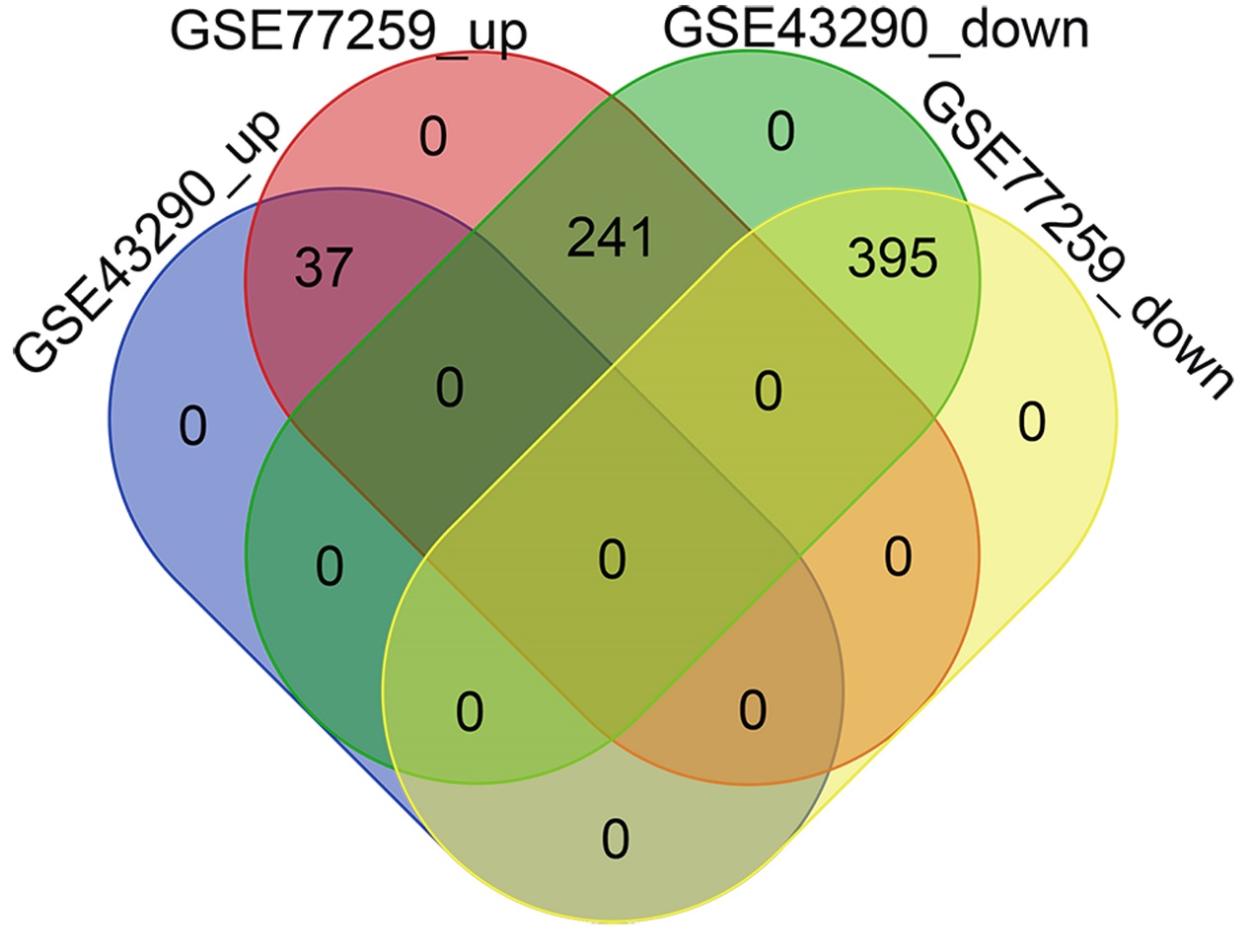

Supplement: Supplementary file 3 — Additional file 3: Supplementary Fig. 1. Venn plot shows the common genes in the two datasets [file 12885_2021_8931_MOESM3_ESM.docx]
