# Supplementary material for: Identification of key genes and pathways associated with resting mast cells in meningioma
Source: BMC Cancer. 2021 Nov 12;21:1209. doi: 10.1186/s12885-021-08931-0 (PMC8590208; doi:10.1186/s12885-021-08931-0)
Supplement: Supplementary file 4 — Additional file 4: Supplementary Fig. 2. WGCNA revealed gene co-expression networks. (A) Analysis of the scale-free fit index for various soft thresholding powers (Left) and analysis of the mean connectivity for various soft-thresholding powers (Right). (B) Network heatmap plot in the co-expression modules. [file 12885_2021_8931_MOESM4_ESM.docx]

**Supplementary figure 2** WGCNA revealed gene co-expression networks.


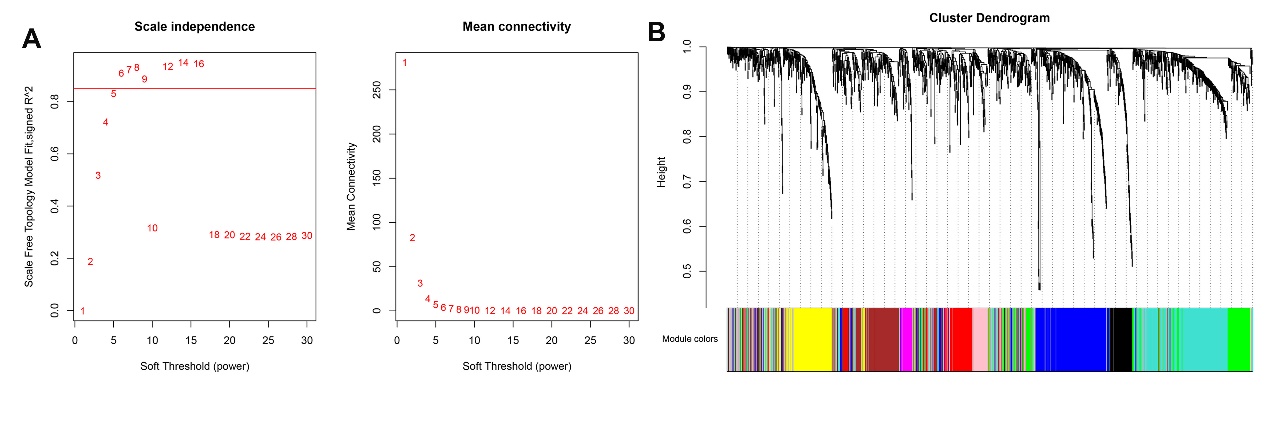


(A) Analysis of the scale-free fit index for various soft thresholding powers (Left) and analysis of the mean connectivity for various soft-thresholding powers (Right). (B) Network heatmap plot in the co-expression modules.
