# Supplementary material for: Identification of key genes and pathways associated with resting mast cells in meningioma
Source: BMC Cancer. 2021 Nov 12;21:1209. doi: 10.1186/s12885-021-08931-0 (PMC8590208; doi:10.1186/s12885-021-08931-0)
Supplement: Supplementary file 5 — Additional file 5: Supplementary Fig. 3. Expression of genes verified by an external dataset GSE54934. (A) Boxplot shows the expression of the nine genes in prognostic risk model between meningeoma and normal samples; (B) Boxplot shows the expression of the 27 transcription factors between meningeoma and normal samples. [file 12885_2021_8931_MOESM5_ESM.docx]

**Supplementary figure 3** Expression of genes verified by an external dataset GSE54934.


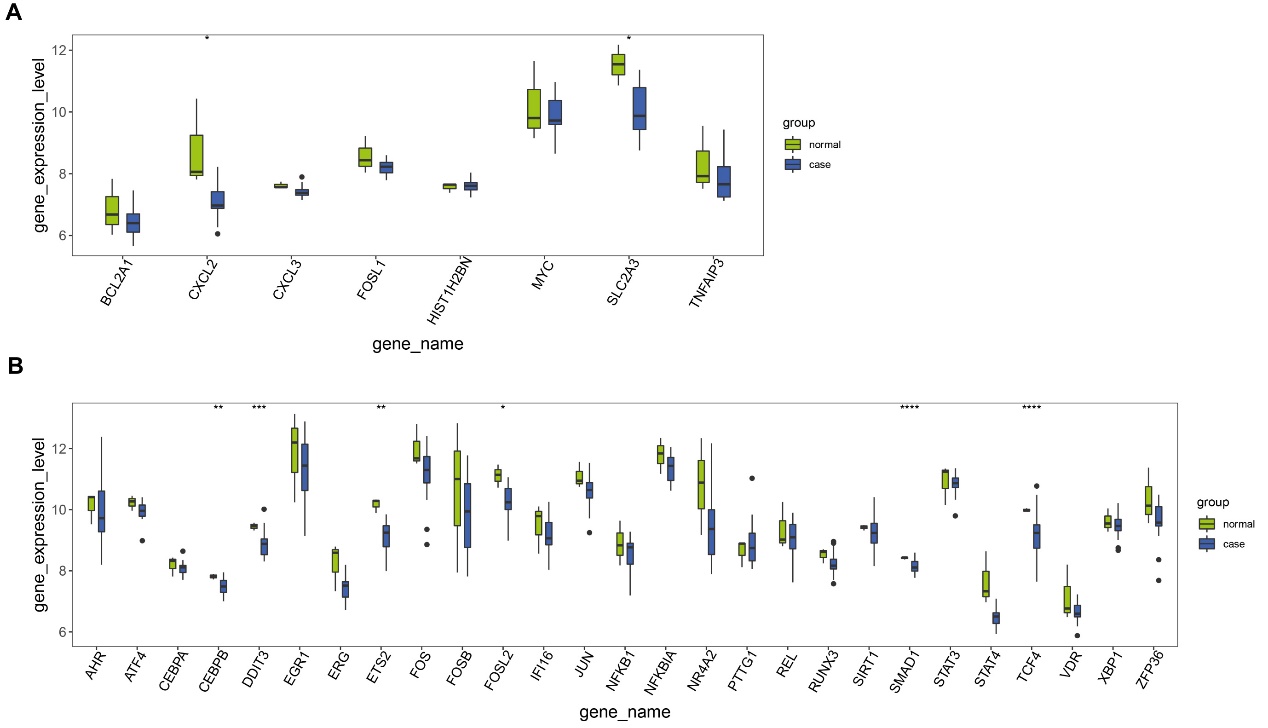


(A) Boxplot shows the expression of the nine genes in prognostic risk model between meningeoma and normal samples; (B) Boxplot shows the expression of the 27 transcription factors between meningeoma and normal samples.
